# Surgical Management of Severe Maxillary Atrophy Using Customized Subperiosteal Titanium Implants: Report of Two Clinical Cases With 42 Months of Follow‐Up

**DOI:** 10.1155/crid/6616749

**Published:** 2026-08-05

**Authors:** Erick Díaz Campos, Franklin Mora Salazar, James R. Collins, Erick Mota

**Affiliations:** ^1^ Biblical Clinic Hospital San Jose, San Jose, San Jose Province, Costa Rica; ^2^ Department of Periodontology, Pontificia Universidad CatÓlica Madre y Maestra-Campus, Santo Domingo, Dominican Republic

**Keywords:** atrophic maxilla, dental implant loading, immediate, subperiosteal implants, titanium

## Abstract

Advanced imaging and direct metal laser sintering enable fabrication of patient‐specific titanium meshes with precise anatomical adaptation, improving stability, surgical predictability, and rehabilitation outcomes in edentulous patients with severe atrophic arches. This article evaluates the long‐term clinical outcomes of customized subperiosteal implants (CSIs) for the rehabilitation of severely atrophic maxillae. Two patients with Cawood and Howell Classes V and VI edentulous maxilla atrophy were treated using customized subperiosteal titanium implants. Implant design and surgical planning of CSIs were performed using cone‐beam computed tomography and intraoral scanning, followed by virtual planning and additive manufacturing. A patient‐specific three‐dimensional printed surgical guide was used to facilitate bone reduction and implant positioning. Following implant placement, an immediately loaded fixed provisional prosthesis was delivered. After a healing period of 4 months, a definitive fixed prosthesis was connected to the subperiosteal implants. Clinical outcomes were assessed over a 42‐month follow‐up period, focusing on soft tissue health, prosthetic performance, and biological or mechanical complications. Both patients demonstrated favorable esthetic and functional outcomes throughout the follow‐up period. No postoperative, biological, or mechanical complications were observed. Implant stability and peri‐implant tissue conditions remained satisfactory, with no evidence of adverse clinical findings. The findings of these case reports indicate that CSIs may represent a reliable long‐term alternative to regenerative procedures in patients with severe maxillary atrophy. This approach allows immediate implant loading and offers a potential treatment option for the fixed prosthetic rehabilitation of edentulous patients.

## 1. Introduction

Dental implant therapy represents a well‐established and widely adopted approach for the rehabilitation of completely edentulous patients, demonstrating high survival and success rates in both medium‐ and long‐term clinical evaluations [1]. Nevertheless, in cases involving severely atrophic maxillary or mandibular arches, the available bone volume is frequently insufficient to permit the placement of conventional endosseous implants. Under such circumstances, reconstructive procedures based on bone grafting techniques are often considered as a prerequisite for implant‐supported rehabilitation [2]. However, these techniques can be complex and invasive, require longer treatment, and carry a risk of intraoperative and/or postoperative complications [3, 4]. Several surgical strategies have been proposed to address severe alveolar bone deficiency and enable implant placement. These approaches include onlay and inlay bone grafting [5, 6], guided bone regeneration using absorbable or nonabsorbable membranes [7], ridge expansion techniques [8], distraction osteogenesis [9], and maxillary sinus augmentation procedures [10, 11]. Although these methods may yield favorable outcomes, they are frequently associated with increased surgical complexity, prolonged treatment duration, and a higher incidence of intraoperative and postoperative complications. When treating upper arches, zygomatic implants can be used, as they have good clinical results and allow immediate loading [12]. However, zygomatic implants have also been associated with several complications, some of which can be quite difficult to manage [12].

Subperiosteal implants (SIs) were first introduced in the early 1940s for treating edentulous maxillary and mandibular arches with severe bone atrophy [13]. However, they have lost relevance among medical professionals due to their insufficient clinical outcomes, high infection rates, complications, and difficulties in placing implants [13, 14].

Over the past two decades, the integration of digital technologies has profoundly transformed the field of implant dentistry [15]. Advances such as cone beam computed tomography (CBCT), intraoral scanning systems, computer‐aided design and manufacturing software, and contemporary additive manufacturing techniques have enabled a higher degree of precision in implant planning and placement [16]. In particular, three‐dimensional visualization combined with direct metal laser sintering allows for the fabrication of patient‐specific SIs that closely conform to individual anatomical characteristics, potentially reducing the risk of mechanical and biological complications [17, 18].

Current research confirms that modern custom SIs show acceptable short‐term outcomes, particularly in cases of severe jaw atrophy. In a recent systematic review, Anitua et al. [18] reported a short‐term survival rate of 97.8% at 21.4 months. Other studies have indicated that well‐designed SIs (e.g., CAD/CAM customization, properly placed abutments), along with enhanced patient care, are essential for achieving long‐term success [19–21]. Currently, the availability of modern manufacturing techniques allows the reconsideration of past techniques, such as SIs, and enables their use in a modern and digital way. This surgical technique offers new solutions for patients with severe bone atrophy or poor bone quality, as in the case of certain systemic diseases such as osteoporosis, diabetes mellitus, and rheumatoid arthritis, or a local oral pathology, such as severe periodontitis or peri‐implantitis [22]. However, further long‐term clinical studies are required to fully validate their efficacy in terms of clinical outcomes (such as soft tissue issues and infection), radiographic changes, and patient satisfaction [18]. This underscores the need for further investigation to establish the medium‐ and long‐term success rates and clinical outcomes.

The aim of these case reports was to present the long‐term results and clinical application of virtual surgical planning (VSP) and custom‐made 3D‐printed subperiosteal titanium implants for fixed prosthetic rehabilitation in patients with severe upper jaw atrophy.

## 2. Cases Presentation

**Figure 1:**
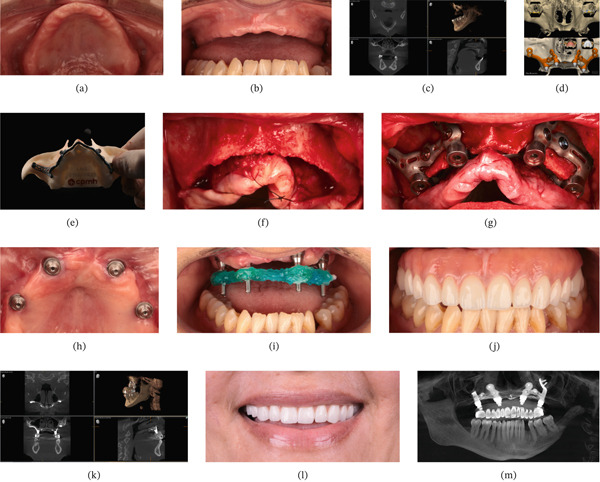
Clinical presentation of Case 1. (a) Occlusal view of Patient 1. (b) Intraoral frontal view of the initial situation of Patient 1. (c) The CBCT scan of Patient 1 revealed an important lack of alveolar bone in all maxillary regions. (d) Virtual planning of the subperiosteal implant and three‐dimensional reconstructions of the subperiosteal implant design for the upper jaw. (e) The fabricated CSI model was screwed to a printed skull for evaluation. (f) Elevation of the mucoperiosteal flap. (g) Subperiosteal implant placed in Patient 1. (h) After 4 months of healing, the temporary immediate restorations were removed. (i) The splinted impression technique was used to accurately transfer the implant position from the mouth to the definitive cast. (j) Intraoral frontal view of the final delivery of the prosthesis. (K) Baseline control radiography. (l) Intraoral view of the final prosthesis after 42 months of follow‐up. (m) Panoramic radiography image showing the status of the implant.

**Figure 2:**
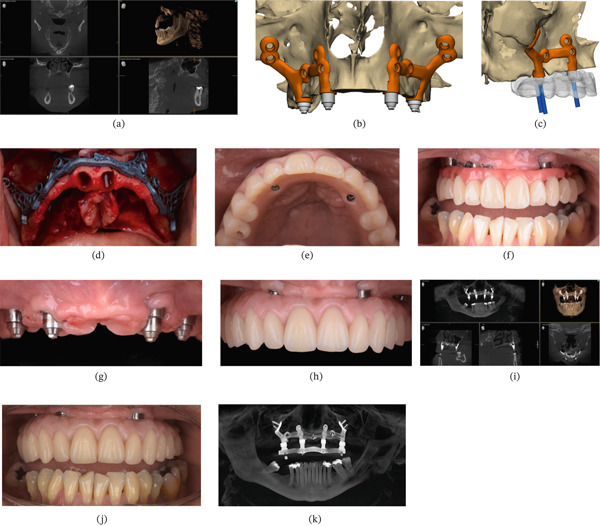
Clinical presentation of Case 2. (a) CBCT scan of Patient 2 showing a severely atrophic edentulous maxilla. (b and c) Three‐dimensional reconstructions of the subperiosteal implant design for the upper jaw and digital planning. (d) Mucoperiosteal flap incised and elevated and custom‐made cutting guide in place using 2.0 mini screws. (e) Occlusal view of the temporary prosthetic immediately screw‐retained prosthesis based on a 3D‐printed design. (f) Frontal view of the immediate temporary prosthetic screw‐retained prosthesis in Patient 2. (g) Temporary immediate restorations were removed after healing for 4 months. (h) Intraoral frontal view of the final prosthesis. (i) CBCT was performed to evaluate the accuracy of the prosthesis stability and the surrounding bone. (j) Intraoral clinical image of the definitive prosthesis at the 42 months follow‐up. (k) Panoramic radiograph of the patient taken at follow‐up.

The first patient was a 65‐year‐old female with an edentulous maxilla (Figure 1a,b). The patient presented with complete nonfunctional mucosa‐supported rehabilitation of the upper arch, which was placed more than 10 years ago. The patient′s dental prosthesis exhibited mobility and poor retention, altering her masticatory function and quality of life. The second case involved a 53‐year‐old female patient who presented with a removable partial prosthesis with mobility and no occlusal stability. Additionally, the patient complained about her appearance, mastication, and speech. According to the Cawood–Howell classification [23], the patients were classified as Grades V and VI (Figure 1c and Figure 2a). Both patients had good systemic (ASA I) and oral health and maintained acceptable oral hygiene habits. Given the severe degree of bone resorption in both cases, the fabrication of CSIs was proposed and accepted by the patients, who signed informed consent forms.

In these cases, an immediate provisional prosthesis, screw‐retained on the same surgical procedure, was planned. Intraoral photographs were taken, and the patients′ prostheses were used for the different planning evaluations. Strategic perforations were made in the prosthesis at the center of the posterior teeth and the cingulum of the anterior teeth using a fissure tungsten laboratory bur to detect the occlusal plane and longitudinal axis of the teeth by CBCT, and a double‐scan technique was used. Afterward, scans were obtained using CBCT (Promax 3D, Planmeca, Helsinki, Finland) of both the prosthesis and the patient, with their prosthesis stabilized with a silicone bite registration (Elite HD Regular Body Zhermack. S.p.A. Badia Polesine [RO], Italy). In addition, an intraoral scan (Primescan, Dentsply Sirona, Germany) of the patient′s prosthesis was performed. Consequently, data from the patient were used to evaluate the most suitable design and positioning for the SIs, and a custom subperiosteal sintered structure in titanium (Ti‐6L‐4V) (Customlife) was designed with four MultiUnit‐type connections (Branemark, Nobel Biocare) and fixations using osteosynthesis screws (CPMH, Brazil) in the areas of greatest density and volume of the malar bone and the upper jaw. Digital implant design was performed using Materialise Mimics software. The fabricated SIs was then attached to a printed skull model for testing purposes (Figure 1d,e and Figure 2b,c). A prosthodontist evaluated several key factors, such as interocclusal clearance, abutment angle, and abutment position.

### 2.1. Surgical Intervention

For both patients, surgical interventions were performed under conscious sedation and local anesthesia (articaine 4% with epinephrine (1:200.000)). After infiltration, a crestal incision was made using a #15 scalpel, and a full‐thickness mucoperiosteal maxillary flap was elevated to provide adequate access for visualization and instrumentation. The dissection limits were the cranial infraorbital ridges, malar bodies laterally, and the anterior half of the hard palate caudally. A custom‐cut 3D‐printed guide (CPMH, Brazil) was also used for predrilling the fixation screw holes and alveolar reduction so that the CSIs were fully in direct contact with the bone. Bone reduction was performed using a piezoelectric handpiece (Woodpecker, Guilin National High‐Tech Zone, Guilin, Guangxi China). The implant was then placed, and the mesh was fixed with 2.0 or 2.4 mm self‐tapping osteosynthesis screws on the nasal and zygomatic buttresses (CPMH digital, Sau Paulo, Brazil). Before the surgery was completed, straight multiunit abutments were placed. A periosteum‐release incision was made for flap passivation to cover the whole section and achieve first‐intention healing (Figure 1f,g). The flaps were closed using a 5/0 nonabsorbable monofilament suture (Techsuture, Rua Jorge Pimentel, Bauru/SP, Brazil). Immediately after surgery, implant position recordings were obtained using photogrammetry to fabricate an immediate prosthesis using subtractive PMMA technology. The restoration was performed 24 h after surgery using an ICam 4D (Imetric 4D Imaging System Sàrl, Courgenay, Switzerland) (Figure 2d–f). The surgical procedure was uneventful, and the patient tolerated the procedure well. For the postoperative period, patients were instructed to take oral antibiotic therapy (amoxicillin/clavulanic acid 1 g/8 h) for the first 7 days, along with analgesics, anti‐inflammatory drugs, and 0.12% chlorhexidine mouthwashes, two or three times a day during the first week. Patients were monitored on a regular basis to identify any issues associated with soft tissue coverage, prosthetic problems, or component failure.

### 2.2. Prosthesis

After 4 months of healing, the patients returned to begin their final rehabilitation without experiencing any biological or prosthetic‐related issues. The temporary immediate restorations were removed from both patients, and splinted impression transfers (Elite HD+, Zhermack SpA, Badia Polesine, Italy) were placed. A conventional impression was obtained with the addition of silicone to fabricate a hybrid prosthesis using a Toronto bar with customized zirconia crowns and light‐curing pink castable resin (NesCem, Ivoclar Vivadent AG, Schaan, Liechtenstein) to simulate the gingiva. Esthetic and functional trials were then performed before the final prosthesis was delivered. Similar procedures were performed in both cases (Figure 1h–k and Figure 2g–i).

During the follow‐up period, no implants were lost, for a survival rate of 100%, and the patient reported good function and esthetics, and a significant improvement in quality of life was observed. CT scans showed after 42 months of final prosthesis fitting that there was no bone resorption around the screws or any ongoing pain, discomfort, or infection, indicating the CSIs were adequately integrated and stable in the surrounding tissues. (Figure 1l,m and Figure 2j,k). Table 1 summarizes the main clinical characteristics, treatment, and outcomes of the presented cases.

**Table 1:** Clinical characteristics, treatment, and outcomes of cases.

Case	Patient age/sex	Clinical condition	Cawood–Howell class [24]	Treatment	Follow‐up	Outcomes	Complications
1	65‐year‐old female	Edentulous maxilla with nonfunctional prosthesis (> 10 years), poor retention and mobility	VI	Customized subperiosteal implant (Ti‐6Al‐4V) with four multiunit connections, fixation with osteosynthesis screws	42 months	No peri‐implant radiolucency adjacent to framework or screws, improved function, esthetics, and quality of life	None reported (biological or mechanical)
2	53‐year‐old female	Partial edentulism with unstable removable prosthesis, impaired mastication, speech, and esthetics	V	Customized subperiosteal implant (Ti‐6Al‐4V) with four multiunit connections, fixation with osteosynthesis screws	42 months	No peri‐implant radiolucency adjacent to framework or screws, functional and esthetic improvement; high patient satisfaction	None reported (biological or mechanical)

## 3. Discussion

This study presents two cases of severe maxillary jawbone atrophy. Both patients lost teeth at a young age due to caries and periodontitis. In this scenario, the patients required extraoral bone grafts to provide the necessary bone support for endosseous implants due to significant alveolar bone loss; however, both patients declined to undergo these treatments. This report details the procedures and methodologies employed in creating and delivering CSIs for patient restoration.

Bone atrophy in edentulous jaws is a major drawback for endosseous implant placement. The most widely used classification of bone atrophy, according to its severity and pattern of bone resorption, is that of Cawood and Howell [24]. The treatment of severe atrophy (Types V and VI) is complicated and often requires the use of large bone grafts, such as those from the iliac crest or skull, which often have unpredictable results and increase morbidity, in addition to requiring months of waiting before implant placement [25].

In this study, we treated only edentulous patients; in particular, we focused on the rehabilitation of the maxilla. Our patients had positive clinical outcomes when subperiosteal custom‐made implants were used. Moreover, patients reported an increase in quality of life and confidence, allowing them to perform basic functions such as chewing and speaking. The patients were also satisfied with the esthetic results of the restorations. Recent consensus reports suggest that customized SIs constitute a promising therapeutic alternative for edentulous patients presenting with severe jaw atrophy. These studies have highlighted favorable short‐term survival rates, a low incidence of major complications, and reduced morbidity when compared with extensive bone grafting procedures [23]. Nevertheless, the ongoing evolution of digital workflows and manufacturing technologies requires professionals to continuously update their knowledge and acquire new skills to remain competitive in the profession. In addition, these implants should be used when conventional implants cannot be placed or when patients refuse to undergo complex bone regeneration techniques.

Although CSIs was not used in patients with cancer in our study, rehabilitation with CSIs has been proposed as an alternative for oncologic patients with atrophic maxillae. Carretero et al. [26] evaluated the survival, clinical success rate, and quality of life of four patients with segmental maxillary defects that had been reconstructed with customized subperiosteal titanium maxillary implants (CSTMIs) through VSP, STL models, and CAD/CAM titanium mesh. The authors reported that CSTMIs can serve as a secure option for reconstructing maxillary defects, enabling the simultaneous rehabilitation of dental structures while also restoring midface projection. However, minor complications linked to SI, such as soft tissue recession accompanied by inflammation, may develop because of inadequate soft tissue coverage but can be addressed via careful patient selection and prompt treatment. Complications such as ascending infections and mechanical issues (e.g., fractures of the implant component) are uncommon [27, 28]. In the case of zygomatic implants, the emergence is often too palatal, which complicates the stability, hygiene, and phonetics of the definitive prosthesis. Moreover, previous chronic sinus pathology increases the zygomatic implant complications, even with the extrasinus technique [29]. For this reason, new technologies in titanium additive printing and 3D planning emerged as a predictable option to restore CSIs in patients who presented with severe maxillomandibular atrophy. Recently, Van den Borre et al. [30] conducted a multicenter study to investigate patient‐ and clinician‐reported outcomes of additively manufactured subperiosteal jaw implant (AMSJI) in the maxilla. Fifteen consecutive patients were included in the study and followed up for 1 year. A survey protocol and clinical and radiographical examination were performed preoperatively (T0) and at 1 (T1), 6 (T2), and 12 (T3) months after permanent upper prosthesis placement. The patients reported an increased oral health‐related quality of life, without complications.

The custom design of SIs allows them to be completely isolated from the maxillary sinus, implying that sinus complications are unlikely to occur. Correct incision design and soft tissue grafting should be implemented to lower the rate of this complication. Patients with atrophic jaws often face a significant challenge because of the insufficient width of keratinized tissues, particularly in the lower jaw area, as noted previously [16]. However, our patients presented with sufficient keratinized mucosa, which reduced the probability of tissue dehiscence and implant exposure. However, most surgeons need enough experience with implants in general to reach the level of expertise required for surgical placement of custom‐made titanium SIs. Moreover, this subject requires further examination, because potential complications may significantly affect the longevity of implant success rates.

Nemtoi et al. [17] conducted a study to investigate the survival rate of a digital technique for the manufacturing of custom‐made SIs and what complications might appear after this type of surgery. The authors demonstrated that the fit of the implants was extremely satisfactory, with a mean rating of 4 out of 5. The mean intervention duration was 86.18 min. Notably, there are no standardized success criteria for evaluating the outcomes of CSIs, and many authors apply established success criteria for osseointegrated implants to CSIs.

The long‐term success of these outcomes highlights the effectiveness of CSIs in achieving both functional rehabilitation and esthetic enhancement. Moreover, a prosthesis‐driven approach eliminates the need for bone grafting and enables patients to recover function immediately via a single surgical operation.

## 4. Conclusions

Within the limitations of the present study, such as the limited patient sample and the relatively short follow‐up, it appears that rehabilitation with CSI using digital planning and CAD/CAM technologies is a viable alternative for the rehabilitation of patients with edentulism and severe alveolar ridge atrophy, allowing immediate loading of the implants. Further research, especially prospective clinical studies with larger patient cohorts and longer follow‐up periods, is needed to confirm these positive preliminary clinical outcomes and thoroughly assess the efficacy of CSIs in the rehabilitation of atrophic jaws.

## Author Contributions

Erick Díaz Campos: conceptualization (lead); investigation (lead); methodology (lead); writing—review and editing (equal). Franklin Mora Salazar: conceptualization (lead); investigation (lead); methodology (lead); writing—review and editing (equal). James R. Collins: writing—original draft (lead); writing review and editing (equal); supervision (lead). Erick Mota: writing—review and editing (equal); visualization (lead).

## Funding

No funding was received for this manuscript.

## Consent

The authors certify that they have obtained all appropriate patient consent forms. In the form, the patient(s) have given his/her/their consent for his/her/their images and other clinical information to be reported in the journal. The patients understand that their names and initials will not be published and due efforts will be made to conceal their identity, but anonymity cannot be guaranteed.

## Conflicts of Interest

The authors declare no conflicts of interest.

## Data Availability

The data that support the findings of this study are available from the corresponding author upon reasonable request.
